# Mutagenicity of Ochratoxin A and Its Hydroquinone Metabolite in the *Sup*F Gene of the Mutation Reporter Plasmid Ps189

**DOI:** 10.3390/toxins4040267

**Published:** 2012-04-16

**Authors:** Steven A. Akman, Marissa Adams, Doug Case, Gyungse Park, Richard A. Manderville

**Affiliations:** 1 Department of Cancer Biology, Wake Forest University Health Sciences, Winston-Salem, North Carolina, NC, USA; Email: madams@wakehealth.edu; 2 Department of Public Health Sciences, Wake Forest University Health Sciences, Winston-Salem, North Carolina, NC, USA; Email: dcase@wakehealth.edu; 3 Department of Chemistry, College of Science and Technology, Kunsan National University, Miryong-Dong, Kusan, Korea; Email: parkg@kunsan.ac.kr; 4 Departments of Chemistry and Toxicology, University of Guelph, Guelph, Ontario, Canada; Email: rmanderv@uoguelph.ca

**Keywords:** ochratoxin A, mutagenicity, DNA adduct, genotoxicity, carcinogenesis

## Abstract

Ochratoxin A (OTA) is a mycotoxin that enhances renal tumor formation in the outer medulla of male rat kidney. Direct DNA damage and subsequent mutagenicity may contribute to these processes. In this study we have determined whether OTA in the absence or presence of activated rat liver microsomes (RLM) or redox-active transition metals (Fe(III) or Cu(II)) causes promutagenic DNA damage in the supF gene of the mutation reporter plasmid pS189 replicating in human Ad293 cells. In addition, we have assessed the mutagenicity of the hydroquinone metabolite (OTHQ) of OTA in the absence or presence of cysteine without added cofactors. Our results show that oxidation of OTA, either by RLM or by transition metal ions, activates OTA to a directly genotoxic mutagen(s). The Fe(III)/OTA system was the most potent mutagen in our experimental system, causing a 32-fold increase in mutant fraction (MF) above the spontaneous control MF. The Cu(II)/OTA system caused a 9-fold increase in MF, while a 6–10-fold increase in MF was observed for OTA in the presence of RLM. The OTHQ metabolite is also mutagenic, especially in the presence of cysteine, in which a 6-fold increase in MF was observed. Our data provide further insight into OTA bioactivation that may account for its *in vivo* mutagenicity in male rat kidney.

## 1. Introduction

**Figure 1:**
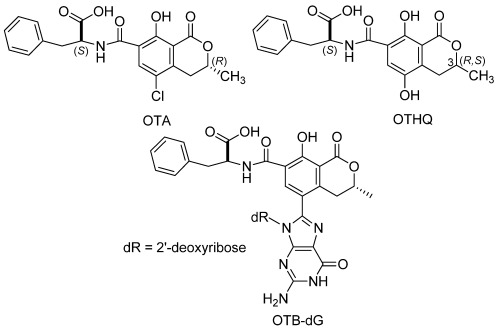
Structure of OTA, OTHQ and the OTB-dG adduct.

Ochratoxin A (OTA, Figure 1) is a human toxin produced by Penicillium verrucosum and several Aspergillus species [1,2]. It is found in a variety of human foods, including cereal grains, e.g., wheat and rye, as well as coffee, beer, and wine [3,4]. OTA has been implicated as a cause of nephropathies and urothelial tract tumors in the Balkans [5,6,7] and in North African countries [8]. The International Agency for Research on Cancer (IARC) has classified OTA as a possible human carcinogen (group 2B) [9], and OTA exposure has been associated with cancer of the urothelial tract in rats [10, and reviewed in 5] and chicks [11]. OTA has also been proposed as a cause of testicular cancer in young men [12,13].

OTA is a substrate for photochemical [14,15,16], electrochemical [17], and transition metal ion-mediated [18,19] oxidation. OTA is also a substrate for enzymatic oxidation by microsomal mixed function oxidases [20,21,22] and enzymes with peroxidase activity [23,24]. Exposure to OTA is cytotoxic to cultured cells [25,26,27,28] as well as *in vivo* in rodent models [29,30,31]. The cytotoxicity of OTA shows a close correlation with the onset of oxidative DNA damage mediated by the toxin through production of reactive oxygen species (ROS) [25,27,28]. 

Studies of OTA-mediated mutagenicity have produced inconsistent results. The original assessment in S. typhimurium in the presence of rat liver post-mitochondrial supernatants was negative [32]. However, De Groene *et al*. observed OTA-mediated mutagenesis in NIH 3T3 cells stably expressing certain P450s [33,34]. Additional studies by the Obrecht-Plumio *et al*. in the presence of high dose OTA (403–1210 µg/plate), kidney microsomes, NADPH, and arachidonate caused the formation of mutagens detectable in S. typhimurium [35]. However, Zepnick and coworkers found negative evidence for OTA-mediated mutagenicity [36]. Palma *et al*. found that OTA-mediated mutagenicity is consistent with oxidative DNA damage and that bioactivation of OTA is not a requirement for the observed mutagenicity [37]. Several other studies have concluded that enzymatic oxidation of OTA does not cause covalent adduction of OTA to DNA [38,39,40] and that mutagenicity stems from by-products of cellular cytotoxicity [41]. However, other recent studies have produced conflicting results that indicate that oxidation of OTA in the presence of nucleosides or DNA yields covalent OTA-DNA nucleoside adducts [42,43,44]. Included in these studies is liquid chromatography-mass spectrometry (LC-MS) results that support the presence of the nonchlorinated OTB-2*′*-deoxyguanosine (dG) adduct (Figure 1) in male rat kidney [44]. Hibi and coworkers have also demonstrated the mutagenicity of OTA in the target tissue (outer medulla) of male rats [45]. In these studies the gpt delta transgenic rat model was employed and the reporter gene mutation assay showed significantly higher levels of deletion mutations compared to controls using DNA extracted from the outer medulla of male rat. The extracted DNA was also examined for 8-oxo-dG formation derived from ROS generation and no evidence for 8-oxo-dG formation was found and OTA exposure failed to increase the frequencies of GC: TA transversion mutations, which are characteristic of 8-oxo-dG-mediated mutagenicity. The *in vivo* mutagenicity assays reported by Hibi *et al*. suggest that oxidative DNA damage does not contribute to OTA-mediated mutagenicity and favor a direct genotoxic mechanism [45].

The positive results on OTA-mediated *in vivo* mutagenicity [45] combined with the finding that OTA generates the OTB-dG adduct in male rat kidney [44] demonstrates that DNA adduction and mutagenicity remains a viable mechanism of action for OTA-mediated renal carcinogenesis [46]. These results prompted us to report the current study, in which we address the mutagenicity of OTA in cell culture, using the human mutation reporter plasmid pSP189 developed by Seidman [47]. The data presented herein indicate that oxidation of OTA, either by microsomal enzymes or by transition metal ions, activates OTA to a directly genotoxic mutagen(s). Synthetic ochratoxin hydroquinone (OTHQ, Figure 1), an OTA metabolite that forms covalent DNA adducts [43], is also mutagenic.

## 2. Experimental Section

### 2.1. Reagents

OTA (≥98%), βNADP, glucose-6-phosphate and glucose-6-phosphate dehydrogenase were purchased from Sigma Chemical Co. (St. Louis, MO, USA). Arochlor^®^-activated rat liver microsomes were purchased from In Vitro Technologies, Inc. (Baltimore, MD, USA). Plasmid pSP189 was received as a generous gift from Dr. Michael Seidman. The construction and properties of pSP189, including its “signature sequence”, have been reported [48]. OTHQ was chemically synthesized as a mixture of diastereomers (3(R/S), Figure 1) using the synthetic protocol previously reported [22] and was ≥96% pure based on reverse-phase HPLC analysis. Stock solutions of OTA and OTHQ (13.7 mM) were prepared in dioxane. Stock solutions containing 10 mM OTA and 5 mM cupric acetate or ferric ammonium citrate (1:2 metal ion:OTA molar ratio) were made in 10 mM MOPS buffer, initially at pH 4, then adjusted to 7.4 with NaOH. Coordination of copper ions by OTA was verified by the appearance of an absorbance peak at 365 nm [18,49]. 

### 2.2. Treatment of Plasmid PSP189 with OTA

In general reactions were carried out in 50 mM potassium phosphate buffer, pH 7.4, except those involving Cu(II)- or Fe(III)-OTA complexes, in which case the buffer was 50 mM HEPES, pH 7.4. For reactions utilizing rat liver microsomes (RLM), a 25 mg/mL microsome suspension and a nucleotide regenerating system consisting of 0.7 mM βNADP, 7.7 mM glucose-6-phosphate, plus 1.5 units/mL glucose-6-phosphate dehydrogenase were pre-warmed to 37 °C for 5 min. All reactions were carried out in 500 µL volumes at 37 °C for 60 min and included 25 µg pSP189, OTA or OTHQ, 625 µg activated microsomes and 125 µL nucleotide regenerating system, where appropriate. Reactions were quenched by cooling on ice, followed by phenol:chloroform:isoamyl alcohol (24:24:1) extraction, chloroform:isoamyl alcohol (24:1) extraction, and ethanol precipitation. Precipitated plasmid was washed twice with 70% ethanol, dried, and dissolved in 10 mM Tris-HCl, pH 8 for analysis.

### 2.3. Transfection of Target Cells

Human Ad293 cells, which are immortal, but not malignant, human cells derived from embryonic kidney, were grown as a monolayer in Dulbecco’s modified Eagle’s medium (Gibco, Grand Island, NY, USA) containing 5% heat-inactivated fetal bovine serum in a humidified 5% CO_2_ atmosphere. Target Ad293 cells, grown to ca. 50% confluence in 100 mm^3^ dishes, were transfected with 10 µg pSP189 DNA by the diethylaminoethyl dextran technique [50].

### 2.4. Mutation Analysis

Low molecular weight DNA was isolated from target Ad293 cells 48 h after transfection by the procedure of Hirt [51]. Plasmid DNA was purified and analyzed for the mutant fraction (MF) as previously described [52]. Briefly, purified plasmid DNA was treated with 0.1 units/µL Dpn I to remove unreplicated plasmid DNA, then used to electrotransform Escherichia coli strain MBM7070. MBMB7070 cells contain an amber mutation in lacZ that is suppressed by wild type supF tRNA, producing functional β-galactosidase. Transformants were plated on Luria broth plates containing 50 µg/mL ampicillin plus isopropyl-β-D-thiogalactoside (IPTG) and 5-bromo-4-chloro-3-indoyl-β-D-galactoside (X-gal, the chromogen). Total (blue + white) and mutant supF-containing (white) colonies are enumerated. Mutant (M) white colonies were confirmed by secondary streaking. 

### 2.5. Statistical Analysis

Poisson regression was used to assess the effect of treatment on the mean number of M colonies. The log of the number of colonies was used as an offset in the models, and pair-wise treatment differences were tested using linear contrasts. The Deviance statistic was used to assess goodness of fit, and a likelihood ratio test comparing the log likelihoods from Poisson and Negative Binomial models was used to test for overdispersion. Weighted means and standard errors (SE) are presented in the text and tables.

## 3. Results

### 3.1. Activation with Rat Liver Microsomes

Exposure to OTA did not significantly enhance the mutant fraction (MF) of plasmid pSP189 replicated in human Ad293 cells above the background value of 3.5 × 10^−6^ (Table 1). Exposure to 0.5–5 mM OTA in the presence of activated rat liver microsomes (RLM) enhanced the MF 6–10 fold to 3.5 ± 0.93 × 10^−5^ (Table 1, *p* < 0.02 for each comparison). However, no dependence of the MF on OTA concentration in the range 0.5–5 mM was discernible. Replacing activated RLM with boiled microsomes totally abrogated the OTA/microsome associated enhancement of mutagenicity.

**Table 1:** Mutagenicity of OTA in the presence of rat liver microsomes.

Expt.	Treatment	Colonies	M Colonies ^a^	MF (×10^4^) ^b^	Mean ± SE
1	Plasmid alone	211267	2	0.09	0.035 ± 0.025
2		81533	0	0.00	
3		274100	0	0.00	
1	1 mM OTA	145467	1	0.07	0.061 ± 0.035
2		48367	1	0.21	
3		295233	1	0.04	
1	RLM + 0.5 mM OTA	165233	3	0.18	0.31 ± 0.087
2		81767	2	0.24	
3		169300	8	0.47	
1	RLM + 1 mM OTA	104167	2	0.19	0.23 ± 0.072
2		57767	2	0.35	
3		275867	6	0.21	
1	RLM + 5 mM OTA	88900	2	0.22	0.35 ± 0.093
2		31333	2	0.64	
3		281700	10	0.36	
1	Boiled RLM + 1 mM OTA	56067	0	0.00	0.032 ± 0.032
2		51567	1	0.19	
3		204900	0	0.00	

^a^ The number of mutant (M) supF-containing (white) colonies; ^b^ Mutant Fraction (MF) = number of M colonies (white)/total (blue + white) colonies.

### 3.2. Mutagenicity of OTHQ

The OTHQ metabolite of OTA can undergo an autoxidative process to generate the quinone electrophile OTQ [22], that reacts with DNA to generate adduct spots, as evidenced by ^32^P-postlabeling [43]. Thus, the OTHQ metabolite of OTA was tested for mutagenicity in the absence of metabolism. Its mutagenicity was also tested in the presence of 1 equiv. cysteine. These experiments were prompted by our earlier findings that the OTQ electrophile reacts covalently with cysteine to form a conjugate that is unstable and undergoes further reactions to afford electrophilic species that may react with DNA [16]. A one hour exposure to 1 mM OTHQ senhanced the MF of plasmid pSP189 replicated in human Ad293 cells 3.6 fold above background to 1.4 ± 0.51 × 10^−4^ (*p* = 0.049, Table 2). Addition of 1 mM cysteine to 1 mM OTHQ further increased the MF to 6.3 fold above background to 2.5 ± 0.68 × 10^−4^ (*p* = 0.003 *vs*. background, Table 2).

**Table 2:** Mutagenicity of OTHQ in the absence or presence of cysteine.

Expt.	Treatment	Colonies	M Colonies	MF (× 10^4^)	Mean ± SE
1	Plasmid alone	40150	2	0.50	0.39 ± 0.22
2		18050	1	0.55	
3		19600	0	0.00	
1	1 mM cysteine	30500	3	0.98	0.73 ± 0.28
2		32700	3	0.92	
3		32700	1	0.32	
1	1 mM OTHQ	12400	4	3.2	1.41 ± 0.45
2		29200	3	1.0	
3		29350	3	1.0	
1	1 mM OTHQ + 1 mM cysteine	11200	6	5.4	2.45 ± 0.60
2		29050	7	2.4	
3		29050	4	1.4	

### 3.3. Mutagenicity of OTA-Transition Metal Ion Complexes

The mutagenicity of OTA in the presence of Fe(III) and Cu(II) was also determined, given that OTA is oxidized by Fe(III) into the quinone electrophile OTQ [22] that provides a rationale for the mutagenicity of OTHQ (Table 2). OTA also reacts with dG in the presence of Fe to generate the OTB-dG adduct shown in Figure 1 [53], and forms an OTA-Fe complex that has been implicated in lipid peroxidation mediated by the toxin [54]. OTA also forms a complex with Cu(II) [18,49] that can facilitate oxidative DNA strand scission [18,19]. Thus, we were interested to determine whether OTA would show mutagenicity in the presence of these redox-active transition metal ions. For these experiments, OTA was kept in a two-fold excess over the transition metal in the non-metal-coordinating HEPES buffer pH 7.4. Both Fe(III) [54] and Cu(II) [18] form 1:1 complexes with OTA and at pH 6.0 the equilibrium binding constant (*K*_1:1_) for Cu(II) is ~2.5 × 10^6^ M^−1^ [18], while a *K*_1:1 _value ~2 × 10^8^ M^−1^ has been determined for Fe(III) binding [54], suggesting complete metal ion coordination by OTA under our experimental conditions. Free Fe chelated to diethylenetriamine-pentacetic acid does produce a small dose dependent increase in MF in the mutation reporter plasmid assay [55]. In these experiments, 5 mM Fe showed a 3-fold increase in MF compared to control, while 0.1 mM Fe failed to enhance the MF [55]. Free Cu(II) has also been shown to act as a mutagen [56]. In our experiments, exposure to 1 mM Fe(III):2 mM OTA enhanced the MF of plasmid pSP189 replicated in human Ad293 cells 32-fold above background to 2.5 ± 0.52 × 10^−4^ (*p* = 0.001, Table 3). Exposure to the 1 mM Cu(II):2 mM OTA complex enhanced mutagenicity to a lesser extent, *ca.* 9-fold above background to 7.1 ± 2.7 × 10^−5^ (*p* = 0.041, Table 3). 

**Table 3:** Mutagenicity of transition metal ion/OTA complexes.

Expt.	Treatment	Colonies	M Colonies	MF (× 10^4^)	Mean ± SE
1	Plasmid alone	12100	0	0.00	0.080 ± 0.080
2		37200	0	0.00	
3		76050	1	0.13	
1	1 mM Cu(II)/2 mM OTA	10067	1	0.99	0.71 ± 0.27
2		50700	3	0.59	
3		38350	3	0.78	
1	1 mM Fe(III)/2 mM OTA	24800	12	4.8	2.52 ± 0.52
2		35600	4	1.1	
3		34650	8	2.3	

## 4. Discussion

There has been considerable debate whether OTA is directly genotoxic [38,39,44,46,57,58]. OTA can promote oxidative DNA damage through ROS generation that causes cytotoxicity [25,27,28] and oxidative DNA damage has been proposed to play an important role in carcinogenicity [41]. However, other lines of evidence favour DNA adduction by OTA [23,42,43,44,46,57]. The *in vivo* mutagenicity findings for OTA do not support oxidative base damage as the initiation event and favour direct genotoxicity in OTA-induced renal carcinogenicity [44]. However, the link between DNA adduction and OTA-mediated mutagenicity has not been firmly established [46]. 

The data presented here demonstrate that OTA is activated to a species that is a directly genotoxic mutagen. The free toxin lacked mutagenicity in the absence of external cofactors, such as RLM (Table 1) or redox-active transition metal ions (Table 3). These findings differ from the mutagenicity data presented by Palma *et al*., which suggested that bioactivation in not required for mutagenicity [37], and are more in line with the data from Tozlovanu *et al*., 2006 showing that DNA adduction by OTA is not observed directly with OTA in the absence of oxidative metabolism [43]. 

That RLM could be used for the conversion of OTA to a genotoxic mutagen in our experimental system (Table 1), suggested that the genotoxic metabolite of OTA is an oxidation product. In this regard, we previously demonstrated that the oxidation of OTA (100 µM) by RLM (1 mg/mL) generated a GSH-conjugate that suggested the intermediacy of the quinone electrophile (OTQ) in the oxidation of OTA [59]. Pfohl-Leszkowicz and coworkers have also outlined a role for GSH in conjugation of OTA-derived electrophiles following bioactivation of the parent toxin [60]. The oxidation of OTA by activated RLM in the presence of a reducing agent (ascorbate) also generates the hydroquinone metabolite OTHQ [59]. OTHQ autoxidizes to OTQ that reacts covalently with DNA to generate DNA adducts [43]; although the structure(s) of such adducts are unknown. This process occurs spontaneously in aqueous media in the presence of molecular O_2_, and no other cofactors are required for conversion of OTHQ into OTQ [22]. While this process has the potential to generate genotoxic ROS, we have recently demonstrated that OTHQ lacks cytotoxicity in mammalian kidney cells, suggesting that the metabolite is ineffective at stimulating ROS production [61]. Thus, DNA damage mediated by OTHQ is mainly due to covalent DNA adduction stimulated by OTQ formation [43]. This background information implies that OTQ formation is likely responsible for mutagenicity stimulated by the RLM/OTA system (Table 1). 

**Figure 2:**
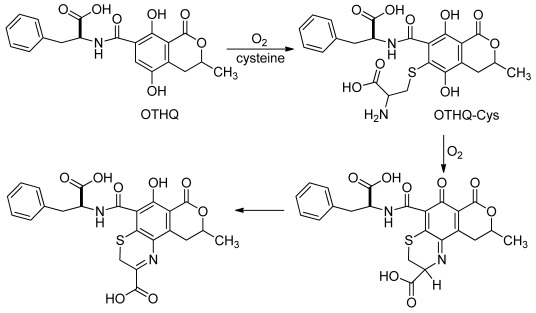
Proposed pathway for reaction of cysteine with OTHQ.

Authentic OTHQ was also mutagen in our experimental system, albeit a weak one (Table 2). The weakness of OTHQ mediated mutagenicity may be a reflection of its slow rate of autoxidation [22]. Prolonged incubation of pSP189 with OTHQ may have enhanced its mutagenicity. However, interestingly, addition of cysteine enhanced the mutagenicity of OTHQ. A possible mechanism for enhancement of OTHQ-mediated mutagenicity by cysteine is suggested by our previous work [16] and outlined by Monks and Lau for the nephrotoxicity of polyphenolic-gluathione conjugates [62]. Thus, as outlined in Figure 2, autoxidation of OTHQ generates OTQ that reacts with cysteine to afford the conjugate OTHQ-Cys [16]. This conjugate is not stable [16] and can also undergo an autoxidative process to reform the quinone structure that reacts covalently with the amine group of the attached cysteine to afford a quinonimine that upon tautomerization will afford the imine electrophile in the new six-membered ring system. The imine functional group forms the basis for DNA adduction by the pyrrolobenzodiazepines, such as anthramycin, that have potential use in anticancer therapies [63]. This proposed pathway may play a role for the heightened mutagenicity of OTHQ in the presence of cysteine (Table 2). 

In our experimental system, Fe(III)/OTA was the most potent mutagen showing a 32-fold MF above background levels (Table 3). This observation may correlate with earlier studies on the reactivity of OTA in the presence of dG to yield OTB-dG [53]. These studies were prompted by reports that OTA forms guanine-specific DNA adducts [64,65] and demonstrated that OTB-dG formation resulted from reaction of OTA/dG in the presence of Fe, Cu, or peroxidase enzymes [53]. Interestingly, Fe generated OTB-dG in the highest yield, ~5 orders of magnitude greater than Cu and peroxidase. The greater reactivity of OTA toward guanine in the presence of Fe, as opposed to Cu, may provide a rationale for the enhanced MF of the Fe/OTA system (Table 3). However, Fe(III) also oxidizes OTA to the quinone electrophile OTQ that reacts with DNA [43] and provides a rationale for the mutagenicity of OTHQ (Table 2). Thus, there are at least two possible mechanisms for Fe(III)/OTA mediated mutagenesis: (1) OTB-dG formation, and (2) OTQ formation with subsequent adduct formation. At present, the DNA adduct(s) formed by OTQ have not been structurally characterized and it is unknown whether the OTB-dG adduct is mutagenic. Our goal is to first characterize the DNA adducts formed by OTQ, and incorporate site-specifically the OTQ adducts and OTB-dG into oligonucleotide substrates so that their biological consequences can be assessed. 

The observation of OTA-mediated mutations in the supF gene of plasmid DNA replicating in human Ad293 cells seemingly is not concordant with the work of Hibi *et al*. [45], who did not observe enhanced mutagenesis in the gpt gene in kidneys of OTA-exposed mice. The explanation for the disparate results is not known; however, methodologic differences may be contributory gpt mutations are detected phenotypically by resistance to 6-thioguanine due to loss of hypoxanthine-guanine phosphoribosyl transferase (HGPRT) catalytic activity. Phenotypically silent gpt mutations that do not affect HGPRT catalytic activity and are therefore not detected in a 6-thioguanine resistance assays may have contributed to underestimation of OTA-mediated mutations in mouse kidney DNA. In contrast, there are no phenotypically silent mutations in the supF gene in the reporter assay used in this study. Hibi *et al*. [45] did observe enhance OTA-mediated production of large (*ca.* 10 kb) deletions in mouse kidney; such mutations are not observable in the plasmid pSP189-based assay. The occurrence of large deletions in the mouse kidney *vs*. base substitutions and frameshift mutations in the *in vitro* plasmid based assay may reflect differences in the OTA activation pathways to a proximate mutagen. Base substitution and/or frameshift mutations mediated by OTA *in vitro* are enhanced by conversion to the hydroquinone in the presence of cysteine or to the phenoxyl radical in the presence of Fe(III), both of which produce DNA base-reactive OTA intermediates. It is possible that OTA activation pathways in the mouse kidney generate phosphodiester backbone reactive intermediates, producing double strand breaks that lead to large deletions, rather than DNA base reactive intermediates. In this regard, differences in OTA activation pathways in the kidney as compared to those utilized in the *in vitro* mutation reporting system may be attributable to the fact that liver, as opposed to kidney, microsomes were used for *in vitro* activation. Future experiments using the *in vitro* mutation reporting system described herein will focus on the impact of OTA bioactivation by kidney microsomes, enabling comparison with the mutagenicity of liver microsome-activated OTA *in vitro* and the *in vivo* OTA-mediated genetic alterations described by Hibi *et al*.
